# Smartphone use in Neurology: a bibliometric analysis and visualization of things to come

**DOI:** 10.3389/fneur.2023.1237839

**Published:** 2023-11-22

**Authors:** William O. Tatum, Emily K. Acton, Brin Freund, Manuel de la Cruz Gutierrez, Anteneh M. Feyissa, Tara Brigham

**Affiliations:** ^1^Department of Neurology, Mayo Clinic, Jacksonville, FL, United States; ^2^Departments of Biostatistics, Epidemiology, and Informatics, University of Pennsylvania, Philadelphia, PA, United States; ^3^University of Pennsylvania Libraries, Philadelphia, PA, United States; ^4^Mayo Clinic Libraries, Mayo Clinic, Jacksonville, FL, United States

**Keywords:** Neurology, smartphone, bibliometric, publication, diagnosis

## Abstract

**Background and objectives:**

Smartphones are a ubiquitous part of society with increasing use as a healthcare tool. We aimed to analyze the published literature on smartphone usage within the field of Neurology to define the scientific landscape and forecast future research initiatives.

**Methods:**

We performed a bibliometric review of smartphone uses in Neurology based on a search of two Web of Science databases from inception through September 16, 2022. This librarian-guided review was conducted using Bibliometrix for data assessment and visualization. Temporal trends in publications, citation counts, collaborations, and author affiliations were among key metrics evaluated. VOS viewer identified hot spots based on generating co-occurrences and bibliographic coupling mapping.

**Results:**

Our search found 3,920 publications. The U.S. produced the most topic-based publications, collaborating most frequently with U.K., Canada, and China-based authors. The most prolific institutions included Karolinska Institute, University of Sydney, and University of Pittsburgh. *Bioelectromagnetics*, *Stroke*, and *Neurology* were the most cited journals. Rapid growth in scientific production occurred in recent years, including during the COVID-19 pandemic. Hotspots and keyword co-occurrence included telehealth, machine learning, and self-management. Temporal trends reflect transitioning from a focus of initial publications regarding mobile phone safety to more recent application of smartphones as “smart” tools for single modality diagnosis, monitoring, management, and treatment of neurological diseases.

**Discussion:**

There has been rapid expansion of the published literature on smartphone uses in Neurology. Initial focus on smartphones and health risk has shifted to uses for neurological disease diagnosis, detection, and management, with relevance as a global interface for collaboration and clinical practice.

## 1 Introduction

In March 2020 the World Health Organization declared the Coronavirus disease 2019 (COVID-19) a pandemic. Unsurprisingly, this had far-reaching implications on social and work-related function in the way clinicians practice medicine on a global scale. Temporary shut-down adversely affecting use of hospital resources and patient care forced alternative methods of mobile healthcare. These included models designed to manage routine chronic neurological disorders in the worldwide scientific community (1–3). Telemedicine provides an adjunctive form of m-Health that is available, accessible, and acceptable to patients as a means to establish diagnoses and monitor patients with neurological disorders. In parallel, there was a rapid surge in COVID-19-related publications (4–6), which provide a rich pool of information on the practical applications of telemedicine modalities to advance healthcare during this period (7).

Smartphone images and videos have become valuable tools to augment the practice of Neurology and provide a supplemental and complementary approach to history and physical examination (8–10). History and physical examination are foundational for a neurological diagnosis, however, there are limits to its accuracy. Web-based smartphones are now widely available throughout the world and contain increasingly sophisticated software to function as multimodal medical instruments, in addition to serving as a portal for real-time communication, audio- and high-resolution video-recorders, and gateway to the Internet (11, 12).

Bibliometrics are a useful technique to provide quantitative analysis of the available literature (13). The availability of scientific literature databases such as Scopus and Web of Science make large volumes of bibliometric data readily accessible, and bibliometric software such as VOSviewer has enabled graphic display of the data a quick assessment of bibliometric analysis to inform scholarly interests (14). Mathematical and statistical trends in scientific research may be uncovered among authors and co-authorships, type and number of citations, and journal assessment to identify the significance of published works and country-specific contributions on trending and growth to foster future collaboration. Because of the changing landscape incorporating smartphones as medical instruments used in neurological disease, we sought to examine publication trends within medical literature. Our aim for this analysis was to provide a comprehensive bibliometric assessment of trends in the scientific production and publication dynamics associated with smartphone use for the diagnosis, monitoring, and management of neurological disease.

## 2 Methods

### 2.1 Data sources and search strategies

Before we performed a search, we consulted an academic librarian (TB) to ensure the data captured would provide the foundation for our results. Thereafter, we sought to elucidate evolving trends to include as many aspects of smartphone use in Neurology as possible. We expanded our search to incorporate a surplus of key words to allow for the emergence of terminology trends as an integral part of representing the current landscape and speculation on future trends. For the primary analyses, studies were identified developing and running searches in the Science Citation Index Expanded (SCI-EXPANDED) (1975-Present) and Emerging Sources Citation Index (ESCI) (2017-Present) [via the Clarivate Analytics Web of Science (WoS) interface] databases. Search terms included various neurology-focused terms, as well as keywords such as smartphone, mobile phone, and mobile app. All searches for the primary analyses were completed on September 16, 2022. The complete search strategy is listed in the Supplementary Table. All WoS citations were downloaded into a plaintext format. Full bibliographic metadata was imported into Bibliometrix and VOS viewer (14, 15). This included detailed information on documents (e.g., publication dates, journal titles, issues/volumes), authorship (e.g., names, institutions, countries), content (titles, abstracts, authors’ keywords), and citations (e.g., reference lists, number of citations).

Additionally, a secondary analysis was performed. This was based on a pilot version of our search strategy and run on two of the most highly recognized platforms containing a wide variety of data points for publications and citations. The results of this search were employed to facilitate a more in-depth categorization of key themes within the published literature on smartphone uses in Neurology. The pilot search was developed and run by a medical librarian (TB) in the Scopus (1823-Present) [via the Elsevier Scopus website], Science Citation Index Expanded (1975-Present) and Emerging Sources Citation Index (2017-Present) [via the Clarivate Analytics Web of Science interface] databases with searches conducted on June 23, 2022. Further details of this separate search are presented in the Supplementary Figure.

### 2.2 Inclusion and exclusion

Only documents written in English from each database underwent final analysis. Articles that were not pre-defined were excluded from analysis. This included gray literature and pre-printed documents. Original research articles, editorials, data papers, errata, letters, notes, surveys, and topical review articles were included for analysis. The publication types that were excluded from our searches of the WoS interface were meeting abstracts, proceeding papers, book chapters, retracted publications, news items, biographical-items, bibliographies, and retractions (for SCI-EXPANDED database search); and proceeding papers (for ESCI database search).

### 2.3 Bibliometric analysis

Our bibliometric review incorporated a performance analysis and included scientific mapping. These bibliometric techniques were completed in order to highlight the contributions made by research constituents and establish the relationships between them (13). All data analyses and visualizations were conducted using Bibliometrix (version 3.0, The R Foundation, Vienna, Austria) (15), and VOS viewer (version 1.6.18, Leiden University, Netherlands) (14). This software was chosen and used to stratify the datasets and the relationship between networks. Topics of focus in our analyses included publication dates, document sources, keywords, authorships, institutional affiliations, countries of origin, and citations.

### 2.4 Analysis of outcome

The WoS databases were searched for topical information that was then extracted for analysis. The data recovered from published works identified authorship, country-specific information, and institutional sources worldwide with the intent of outlining relationships, timelines, and trends. Terms as keywords in documents were counted only once per item. To analyze chronological reporting, the number of publications released per year was assessed. In order to accurately demonstrate dynamic trends in the release of publications, the annual growth rate was evaluated. This was relative to the number of publications released over the established timeline.

### 2.5 Visualization analysis

Visualization was performed by importing data from our search to construct, display, and highlight bibliometric networks using VOS viewer to analyze relationships. The overall networks serve to highlight relationships between our chosen topics of interest and related subtopics. Spheres within the maps reflect individual elements. The sizes for each sphere are relative to the cumulative number of documents contained. Bigger spheres are comprised of elements that reflect a larger number of documents. Lines connecting the spheres within the networks and their thickness represent the strength of an association between them. The final networks reflect a composite of the data and provide the landscape for a web of relationships between targeted datapoints. Each node represents distinct elements targeting topics of interest (e.g., keywords, countries, etc.).

## 3 Results

**Figure 1:**
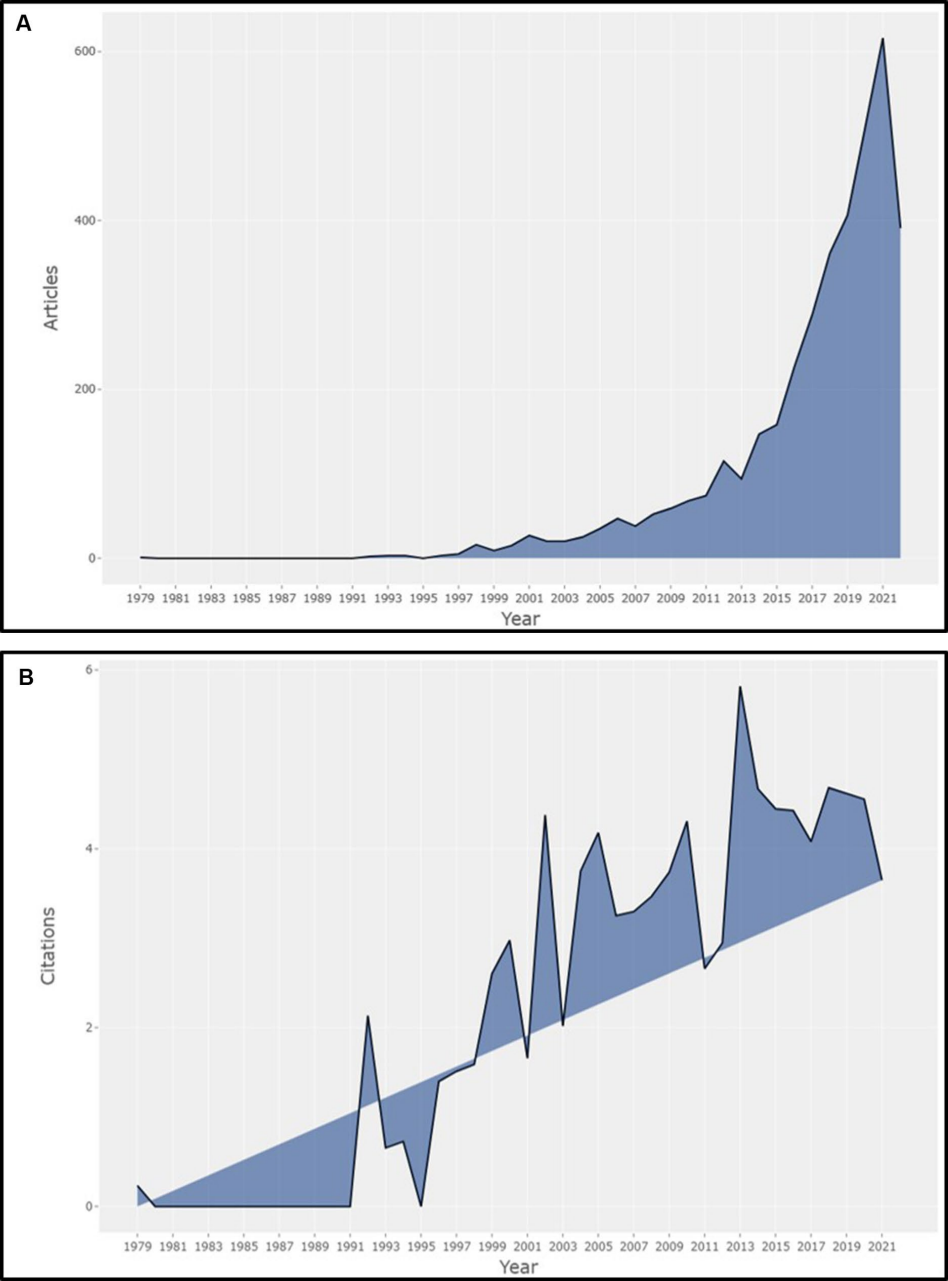
**(A)** Annual scientific production of documents identified by our search for literature on smartphones and Neurology. Of note is panel **(A)** is based on 3,835 of the 3,920 documents included in our Review, as 85 documents did not have the year of publication included in the metadata. **(B)** Average article citations per year for documents identified by the search for literature on smartphones and Neurology revealing overall increasing trends in the mean number of total citations per year for these documents.

The comprehensive search assessing literature on smartphone use in neurology (spanning from database inception through September 16, 2022), yielded a total of 3,920 documents from 18,239 authors and 1,365 sources (i.e., journals, books, etc.). There was a precipitous and steady rise in the number of scientific publications per year incorporated in the bibliometric analyses from one publication in 1979 to 616 in 2021 (Figure 1). The average annual growth rate for the scientific production of the literature was 22.01% over the study time period (16). The average citations per document were 19.38. Overall, there was an increasing trend in the mean number of total citations per year for documents involving smartphone use in Neurology (Figure 1B).

One author (BF) performed a separate secondary analysis including a dataset from the WoS (3751) and Scopus (5237). This was designed to separate documents involving smartphone use in neurological disorders by theme. A flow diagram is present in the Supplementary Figure to reflect this information breakdown. Using a pilot version of the search strategy from our primary analysis (spanning from database inception to June 23, 2022), we identified 8,988 reports. Following de-duplication, we found 6,237 total reports. We excluded 603 for no abstract availability and 3,146 reports that did not include either neurological disorders and/or smartphones resulting in 2,488 reports available for analysis. Of these documents, 1,154 focused on “Use of Smartphones for Diagnosis, Monitoring, and/or Management of Neurological Diseases,” 470 on “Use of Smartphones for Treatment of Neurological Diseases,” 275 on both of the aforementioned themes (i.e., use for “Diagnosis, Monitoring, and/or Management” and “Treatment”), and 589 on “Effects of Smartphones in Causing or Aggravating Neurological Diseases” (i.e., phones causing gliomas).

### 3.1 Keywords

**Figure 2:**
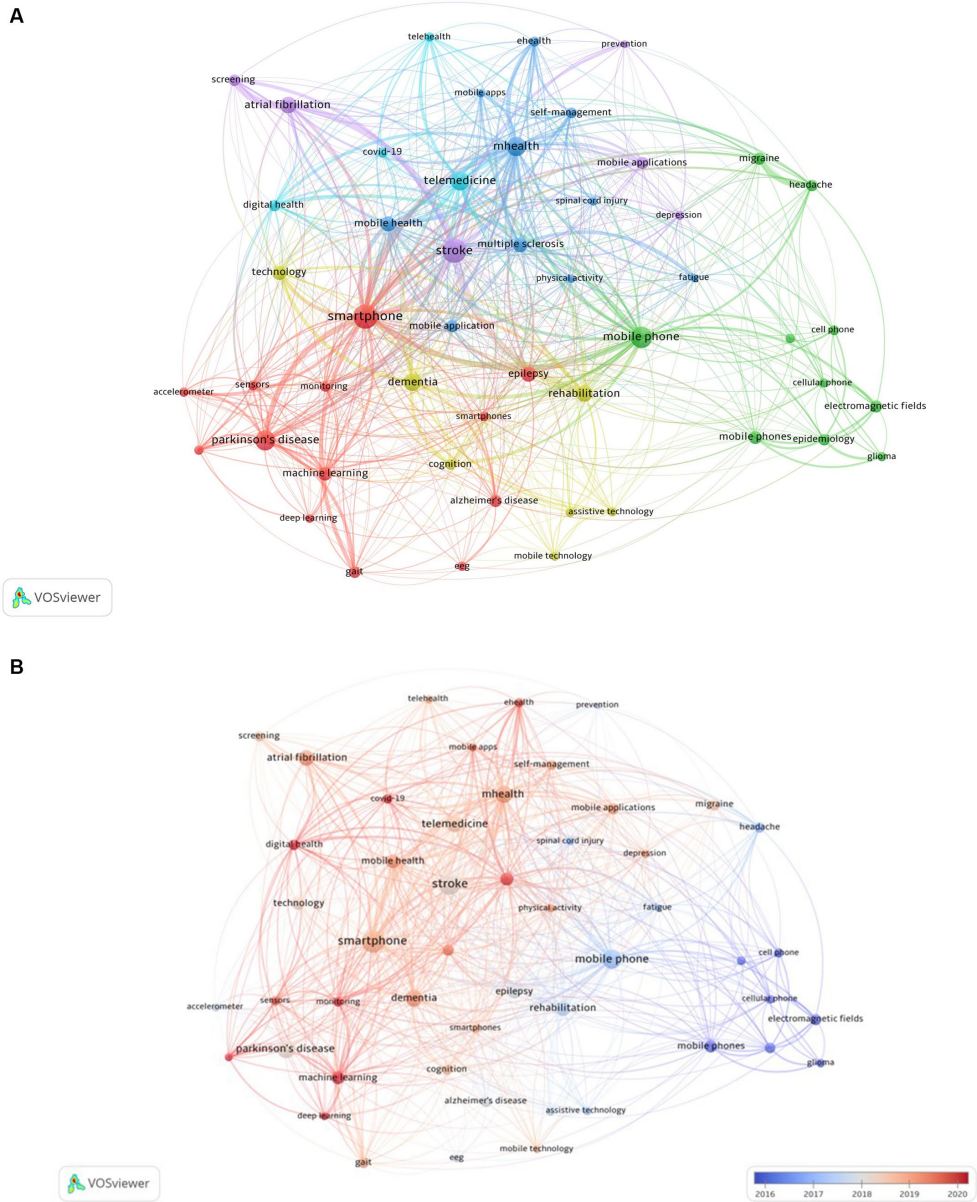
**(A)** Keyword co-occurrence network. Primary colors reflect the clusters of keyword topics. Most frequent neurological diagnoses based on co-occurring keywords were purple = “stroke,” red = “Parkinson’s disease,” yellow = “dementia,” green = “migraine,” light blue = “COVID-19,” and dark blue = “multiple sclerosis.” **(B)** Keyword co-occurrence network. The blue-red color gradient reflects the temporal trends in keyword utilization.

A total of 8,222 author-specified keywords were identified from 1,365 sources published between 1979 and 2022. Of the different neurological conditions, stroke, Parkinson’s disease, and dementia were the most frequently author-generated keyword within the documents focused on Neurology and smartphones (*n* = 247, 178, and 143 occurrences, respectively). Hot topics in the literature on Neurology and smartphones, as well as the relationships between these topics are illustrated via an author-generated keyword co-occurrence network (Figure 2). Further, evolution of hot topics within this pool of literature over time is depicted via an overlay visualization of the publication’s years associated with co-occurring keywords (Figure 2B). This revealed a thematic evolution of author keywords, with the early focus on mobile phone-related safety concerns transitioning to the modern application of smartphones as diagnostic, educational, monitoring, management, and treatment tools within the field of Neurology.

### 3.2 Journals

*JMIR m-Health and u-Health* was the most relevant of 1,365 total sources (*n* = 122 published documents), followed by *Sensors* (*n* = 89), and *PLOS One* (*n* = 59) (Table 1). *JMIR m-Health and u-Health* had an impact reflected by an h-index of 24, g-index of 34, and m-index of 2.4 with 1,656 total citations of the 122 published documents beginning in 2013. *Bioelectromagnetics*, *Stroke*, and *Neurology* were the most cited sources within the reference lists for the document set focused on Neurology and smartphones, with 1,961, 1,894, and 1,841 citations, respectively (Table 1).

**Table 1:** Top 25 relevance and citation metrics for sources, authors, and affiliations.

Most relevant sources	Number of documents	Most cited sources	Number of citations	Most relevant authors (fractionalized)	Number of documents (fractionalized)	Most relevant affiliations	Number of documents
*JMIR MHEALTH AND UHEALTH*	122	*BIOELECTROMAGNETICS*	1961	*HARDELL L*	21.51	*KAROLINSKA INST*	141
*SENSORS*	89	*STROKE*	1894	*CARLBERG M*	13.50	*UNIV SYDNEY*	125
*PLOS ONE*	59	*NEUROLOGY*	1841	*LIN JC*	13.00	*UNIV PITTSBURGH*	104
*JOURNAL OF MEDICAL INTERNET RESEARCH*	55	*PLOS ONE*	1823	*MILD KH*	9.88	*UNIV TORONTO*	93
*BMJ OPEN*	54	*MOVEMENT DISORD*	1393	*KUNDI M*	8.62	*HARVARD MED SCH*	*92*
*BIOELECTROMAGNETICS*	41	*J MED INTERNET RES*	1234	*SCHUZ J*	7.74	*UNIV OXFORD*	89
*FRONTIERS IN NEUROLOGY*	40	*NEW ENGL J MED*	1217	*FEYCHTING M*	7.45	*STANFORD UNIV*	82
*INTERNATIONAL JOURNAL OF ENVIRONMENTAL RESEARCH AND PUBLIC HEALTH*	39	*LANCET*	1203	*HOCKING B*	6.50	*NORTHWESTERN UNIV*	74
*SCIENTIFIC REPORTS*	38	*JAMA J AM MED ASSOC*	1070	*ROOSLI M*	5.05	*SEOUL NATL UNIV*	72
*IEEE ACCESS*	35	*CIRCULATION*	1018	*JOHANSEN C*	4.83	*UNIV MICHIGAN*	72
*JMIR RESEARCH PROTOCOLS*	30	*ARCH PHYS MED REHAB*	981	*MORTAZAVI SMJ*	4.78	*UNIV MELBOURNE*	67
*EPILEPSY & BEHAVIOR*	26	*SENSORS BASEL*	923	*AHLBOM A*	3.64	*DUKE UNIV*	66
*TRIALS*	25	*JMIR MHEALTH UHEALTH*	915	*AUVINEN A*	3.21	*KINGS COLL LONDON*	62
*NEUROPSYCHOLOGICAL REHABILITATION*	23	*AM J EPIDEMIOL*	847	*ALBERTS JL*	3.20	*UNIV CALIF SAN FRANCISCO*	60
*IEEE JOURNAL OF BIOMEDICAL AND HEALTH INFORMATICS*	22	*GAIT POSTURE*	847	*LEE S*	2.91	*MAYO CLIN*	58
*STROKE*	22	*J NEUROL NEUROSUR PS*	718	*TATUM WO*	2.87	*MONASH UNIV*	58
*DISABILITY AND REHABILITATION*	20	*IEEE ENG MED BIO*	712	*MINEN MT*	2.87	*TEL AVIV UNIV*	56
*HEADACHE*	20	*LANCET NEUROL*	704	*PATTERSON V*	2.83	*UNIV WASHINGTON*	56
*ENVIRONMENTAL RESEARCH*	19	*IEEE T BIO MED ENG*	646	*LEE K*	2.80	*UNIV CALIF LOS ANGELES*	53
*APPLIED SCIENCES-BASEL*	18	*RADIAT RES*	606	*LIPTON RB*	2.75	*WASHINGTON UNIV*	53
*AMERICAN JOURNAL OF EPIDEMIOLOGY*	17	*EPILEPSIA*	602	*LEE J*	2.70	*JOHNS HOPKINS UNIV*	52
*ELECTROMAGNETIC BIOLOGY AND MEDICINE*	17	*COCHRANE DB SYST REV*	590	*GUIDETTI S*	2.68	*UNIV HOSP*	48
*INTERNATIONAL JOURNAL OF STROKE*	16	*OCCUP ENVIRON MED*	554	*O’CONNOR S*	2.57	*ALBERT EINSTEIN COLL MED*	46
*JOURNAL OF NEUROENGINEERING AND REHABILITATION*	16	*EPIDEMIOLOGY*	553	*SODERQVIST F*	2.57	*YONSEI UNIV*	45
*JOURNAL OF TELEMEDICINE AND TELECARE*	16	*BMJ BRIT MED J*	544	*REDMAYNE M*	2.56	*INST CANC EPIDEMIOL*	44

### 3.3 Authors and institutions

Of the 18,239 authors listed in the 3,920 documents resulting from our search, 196 single authored documents were written by 155 solo authors. There was a total of 18,084 documents involving more than a single author with an average of 4.65 authors per document, 0.22 documents per author, and 19.38 average citations per document. Table 1 lists the top 25 most relevant authors where Hardell, Carlberg, and Lin were the most prolific authors (fractionized) with 21.5, 13.5, and 13 articles published, respectively. The top author who published the most documents accounted for 1.5% of all recoverable articles. Table 1 lists the top 25 most relevant institutional affiliations with the Karolinska Institute, University of Sydney, and University of Pittsburgh producing 141, 125, and 104 documents, respectively.

### 3.4 Countries

**Figure 3:**
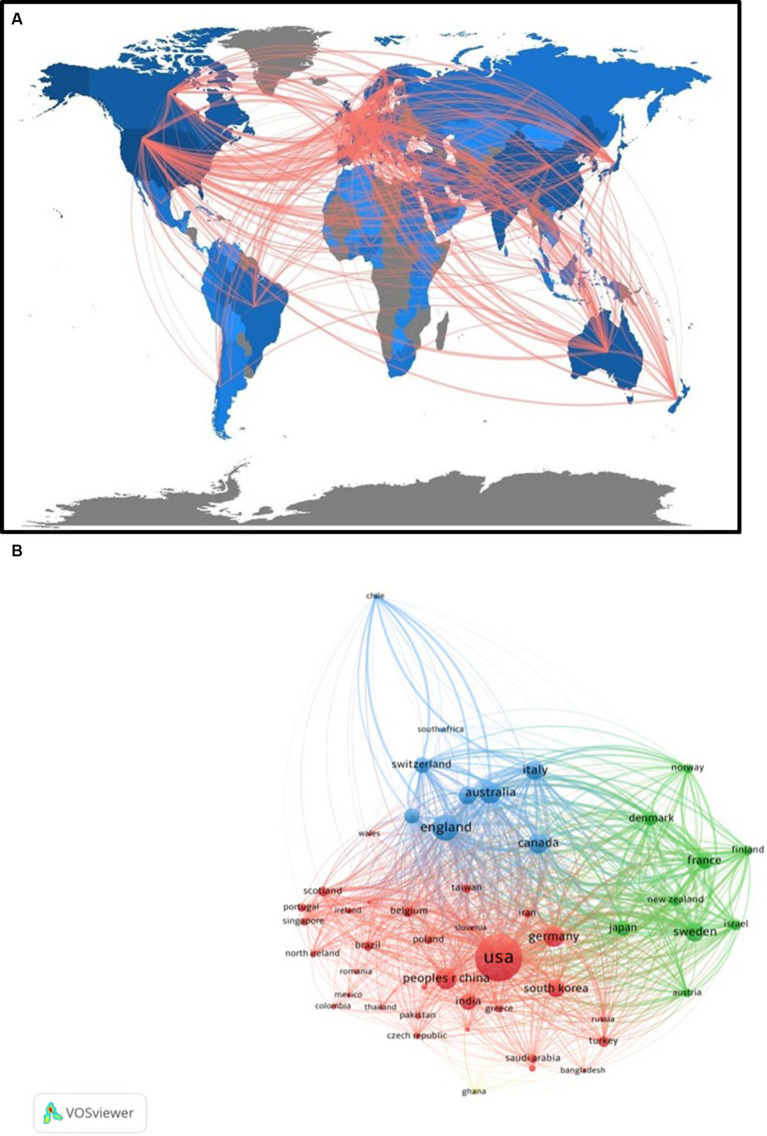
**(A)** Collaboration world map. The red lines indicate inter-country collaboration linkages on documents identified by our search for literature on smartphones and Neurology. The gray-blue color gradient indicates the scientific production by country, with greater numbers of documents indicated by greater intensity of blue coloration. **(B)** Country Bibliographic Coupling network. Primary colors reflect clusters of countries based on shared references within published documents.

The Collaboration World Map illustrates both the scientific production of documents by country and the inter-country collaboration linkages (Figure 3). The countries with the greatest scientific production were the U.S. (*n* = 4,052), U.K. (*n* = 1,175), and Australia (*n* = 854). The U.S. most frequently collaborated on these documents with the U.K., Canada, and China, encompassing 81, 61, and 48 collaborations, respectively, (Figure 3). The Country Bibliographic Coupling network is depicted in Figure 3B, revealing the tendency of these countries to share references within their published documents.

## 4 Discussion

To our knowledge, this is the first study to review the research status of smartphone use in Neurology. Our bibliometric analysis identified changing trends in research production related to smartphone uses in Neurology prior to September 16, 2022, that herald their increasing utilization as medical instruments (Figure 1). The intent of bibliometric analysis is to provide a foundation for future research based on past geographic and interconnected hot spots. By performing a broad-based search regarding research on phone use in neurology, we were able to reveal a binary division in the evolving landscape of smartphone use. We felt it was important to point out the past to outline the shift from safety in the evolution of smartphone use to utilization in neurology to speculate about future trends. Using a variety of bibliometrics tools, we found an increasing number of articles supporting the use of smartphones as an evaluation and management tool to assist physicians in supporting a neurological diagnosis (17–19), facilitate self-learning (20–22), and as a means to assist with reporting and monitoring responses to treatment (23–25). Bibliometrix and VOSviewer were used to represent the metadata obtained from WoS, and to illustrate visual display maps of networks to identify trends in the literature governing smartphone use in neurological disease. As a diagnostic tool, smartphones have been used in Neurology and led to innovations in the longitudinal care of patients for a wide range of disorders including stroke, multiple sclerosis, Parkinson’s disease, sleep disorders, and epilepsy (8, 9, 26). Looking at publications on Neurology and smartphone use over time, research was initially concentrated on brain tumor and safety risks attributed to smartphones (27–29), but transitioned to focus on the application of this technology for disease detection, clinical management, and as a tool for applied medical science more broadly (17–25). Emerging trends have become increasingly evident by elucidating the exponential rise in annual number of publications during the COVID-19 pandemic in developed and developing countries alike. However, the increasing trend for publication in our topical search occurred prior to the pandemic.

Using VOS viewer to generate networks, hotspots were identified for both co-occurring keywords and bibliographic coupling of countries pioneering work in this field. The U.S. is the primary site of published research, though European and Australian institutions (i.e., Karolinska Institute and University of Sydney) were active sites of document publication. Academic institutions with a strong interest in scientific research were primarily involved in most of the published articles. The top institutions involved in smartphone and Neurology publications located in the U.S. were the University of Pittsburgh, Harvard Medical School, and Stanford University. This suggests that despite the ubiquitous nature of smartphones, institutions in developed countries ranked first in reporting influential literature in accord with our bibliometric analysis at this time.

Networks, together with other bibliometric analyses, demonstrated the relationships between specific authors, institutions, and countries where research efforts have been advanced. This could provide a critical guide to form bonds between these groups that could lead to collaborative efforts and the generation of high-quality publications. Keywords summarize and crystallize the essence of the topic evaluated in publications. While initial use of the term “smartphone” was identified in the mid-1990s, we sought to be more inclusive to expand our keywords given that oftentimes the nomenclature of an item evolves over time as it has done with a lexicon now referred to as a smartphone (30). To be as comprehensive as possible, in our bibliometric review, we included keywords that could appear irrelevant but may have included the same description previously in use over time for that specific item. Additionally, to provide comprehensive search results, we attempted to include a wide range of free-text terms or keywords for each of the concepts selected. Our librarian developed the search strategy through an iterative process in which the terms used were modified, based on what was already retrieved. Common keywords were analyzed to highlight hot topics linking smartphone use in Neurology to other research topics in the field. Co-occurring keywords in a cluster analysis revealed new areas of focus including m-Health, machine learning, and self-management within the growing field of telemedicine over recent years.

In a separate secondary analysis, we found most relevant documents were focused on diagnosis, monitoring, and management of neurological conditions with smartphones. The second most common theme among relevant documents was smartphones’ risk of cause or aggravating neurological disease (e.g., glioma). A significant minority focused on treatment, and a lesser number on both diagnosis, monitoring, and management together with treatment. Neurological conditions (e.g., stroke and epilepsy) account for a significant global burden and are among the most common diseases encountered in clinical practice (31, 32). However, access to clinicians is limited in many countries where smartphone use could enhance telemedicine and facilitate collaboration within neurological subspecialties. Therefore, efforts should be made to expand the study of smartphones in diagnosis and treatment of neurological disorders given our findings and the need for improvement in access to care.

Our bibliometric analysis has some limitations. We recognize that only two databases were searched within the WoS “suite” of offerings. There are many more indexes that may have augmented our search, though we identified a high volume of documents and anticipate significant yield from WoS as a database focused on healthcare. Furthermore, as a predictive model our bibliometric review used a quantitative method to identify the number and also variety of document types that may have overly generalized uses of smartphones in Neurology including diagnostics and aggravation of neurological conditions. Doing so may impose limitations on its interpretation on utility based on our search strategy of the literature. Furthermore, despite a thorough and transparent search, we could have inadvertently excluded some sources pertinent to our bibliometric analysis including recently published and emerging documents. We also limited publications to full-text and abstracts written/translated into English which may have filtered out some articles pertinent to our search. Similarly, incorporation of gray literature could have provided a different focus not recovered by reviewing only published literature (33). The timing and effect size created by COVID-19 may also have influenced the trajectory of documents published and blurred the use of smartphones due to the increase in telemedicine during the pandemic. Nonetheless, we also noted an upward trend in publications prior to the pandemic and anticipate ongoing trends given the landscape that is likely to be forever changed as a result. Selected keywords as part of a bibliometric review are fundamental to the analytic process. We acknowledge that our choice of keywords selected in the search strategy could have excluded synonyms that impacted accurate frequency and clustering. However, terms were chosen based on author generated keywords that were present in peer-reviewed documents. Therefore, it is probable that those terms chosen reflected a reliable means to remain inclusive. To that end, one of the co-authors has expertise in library science and carefully reviewed all aspects of the literature search prior to recovery of metadata. While our analysis of the literature involving smartphones was rigorous with respect to trends in scientific publication to outline the intellectual structure of a field by analyzing the social and structural relationships between different research constituents, unlike a meta-analysis, it is unable to address the relative quality of the documents extracted from the search results. Overall, despite its limitations, our bibliometric study highlights the topical focus and reveals areas of interest using smartphones in Neurology. Exponential growth in the number of documents reflects ongoing interest in patients with neurological diseases in parallel with the rise in m-Health.

## 5 Conclusion

In this bibliometric review, we identify evolving trends in the use of smartphones in Neurology. Since the first publication over 40 years ago, the literary playing field involving smartphone usage in Neurology provides insights into continued and future trends in the space of personal electronic devices involving incremental use of smartphones in clinical medicine and applied research as a diagnostic and management tool in the field of Neurology. Further, the evolving landscape incorporates literature from countries across the globe to portray international impact. At present, the U.S. is the most prolific country contributing information with collaboration throughout the globe. As ambulatory and telehealth continues to grow as an active area of research, smartphone use promises improved access to patients with neurological disorders. Areas uncovered by high prevalence keywords in documents associating smartphones and Neurology predict telemedicine, machine learning, and self-management are hotspots for future research. Based on our bibliometric analysis, we anticipate incremental use of smartphones as a vital tele-tool in neurological research and clinical practice.

## Data availability statement

The original contributions presented in the study are included in the article/Supplementary material, further inquiries can be directed to the corresponding author.

## Author contributions

WT, EA, and TB contributed to conception and design of the study. EA, BF, and TB performed the analyses. WT wrote the first draft of the manuscript. All authors contributed to manuscript revision, read, and approved the submitted version.
