# Supplementary material for: Smartphone use in Neurology: a bibliometric analysis and visualization of things to come
Source: Front Neurol. 2023 Nov 22;14:1237839. doi: 10.3389/fneur.2023.1237839 (PMC10703293; doi:10.3389/fneur.2023.1237839)
Supplement: Supplementary file 2 [file Presentation_1.PPTX]

## Slide 1
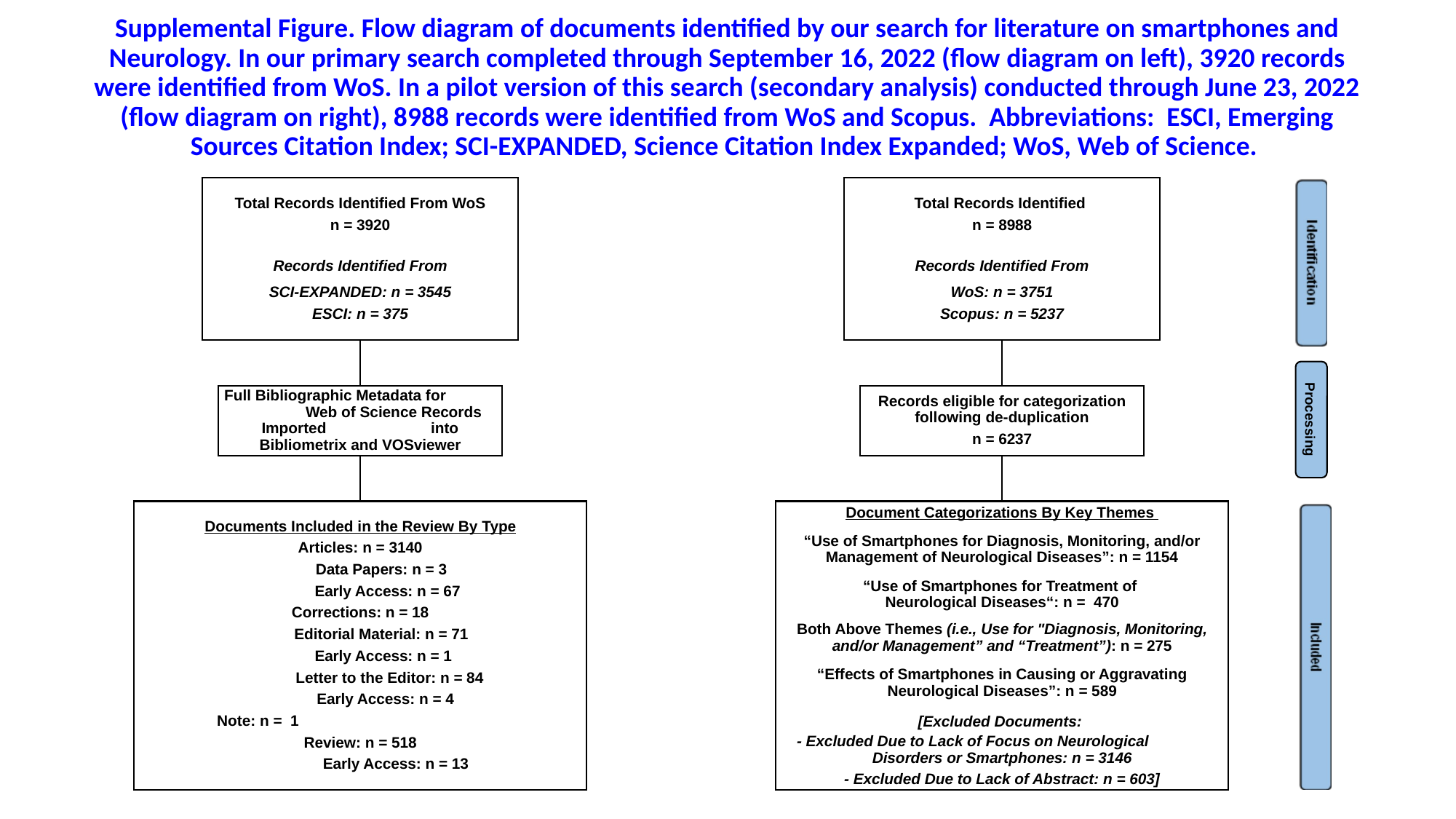

# Supplemental Figure. Flow diagram of documents identified by our search for literature on smartphones and Neurology. In our primary search completed through September 16, 2022 (flow diagram on left), 3920 records were identified from WoS. In a pilot version of this search (secondary analysis) conducted through June 23, 2022 (flow diagram on right), 8988 records were identified from WoS and Scopus. Abbreviations: ESCI, Emerging Sources Citation Index; SCI-EXPANDED, Science Citation Index Expanded; WoS, Web of Science.
Processing
