# Supplementary material for: Smartphone use in Neurology: a bibliometric analysis and visualization of things to come
Source: Front Neurol. 2023 Nov 22;14:1237839. doi: 10.3389/fneur.2023.1237839 (PMC10703293; doi:10.3389/fneur.2023.1237839)
Supplement: Supplementary file 3 [file Presentation_2.PPTX]

## Slide 1
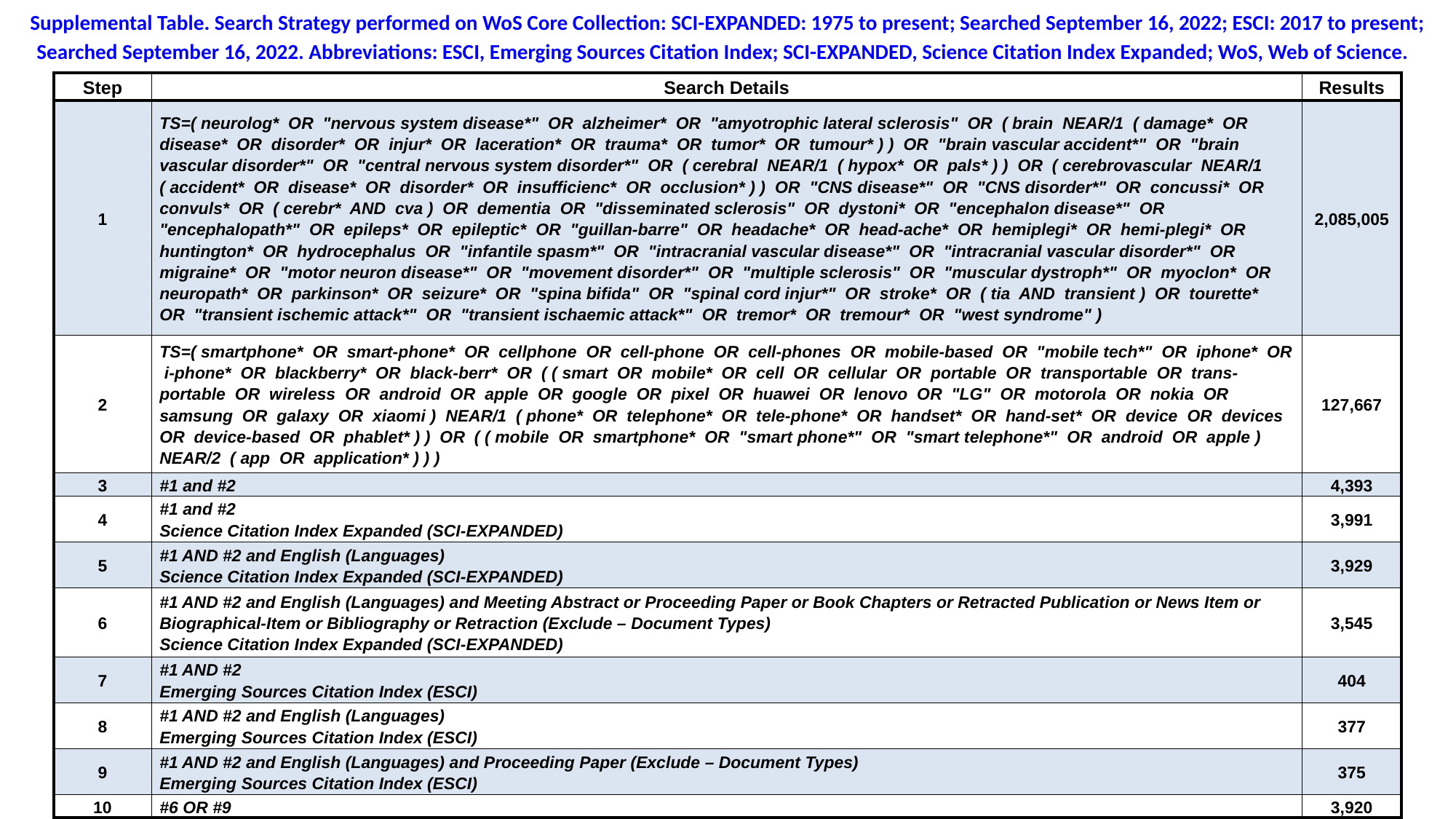

Supplemental Table. Search Strategy performed on WoS Core Collection: SCI-EXPANDED: 1975 to present; Searched September 16, 2022; ESCI: 2017 to present; Searched September 16, 2022. Abbreviations: ESCI, Emerging Sources Citation Index; SCI-EXPANDED, Science Citation Index Expanded; WoS, Web of Science.
| Step | Search Details | Results |
| --- | --- | --- |
| 1 | TS=( neurolog\* OR "nervous system disease\*" OR alzheimer\* OR "amyotrophic lateral sclerosis" OR ( brain NEAR/1 ( damage\* OR disease\* OR disorder\* OR injur\* OR laceration\* OR trauma\* OR tumor\* OR tumour\* ) ) OR "brain vascular accident\*" OR "brain vascular disorder\*" OR "central nervous system disorder\*" OR ( cerebral NEAR/1 ( hypox\* OR pals\* ) ) OR ( cerebrovascular NEAR/1 ( accident\* OR disease\* OR disorder\* OR insufficienc\* OR occlusion\* ) ) OR "CNS disease\*" OR "CNS disorder\*" OR concussi\* OR convuls\* OR ( cerebr\* AND cva ) OR dementia OR "disseminated sclerosis" OR dystoni\* OR "encephalon disease\*" OR "encephalopath\*" OR epileps\* OR epileptic\* OR "guillan-barre" OR headache\* OR head-ache\* OR hemiplegi\* OR hemi-plegi\* OR huntington\* OR hydrocephalus OR "infantile spasm\*" OR "intracranial vascular disease\*" OR "intracranial vascular disorder\*" OR migraine\* OR "motor neuron disease\*" OR "movement disorder\*" OR "multiple sclerosis" OR "muscular dystroph\*" OR myoclon\* OR neuropath\* OR parkinson\* OR seizure\* OR "spina bifida" OR "spinal cord injur\*" OR stroke\* OR ( tia AND transient ) OR tourette\* OR "transient ischemic attack\*" OR "transient ischaemic attack\*" OR tremor\* OR tremour\* OR "west syndrome" ) | 2,085,005 |
| 2 | TS=( smartphone\* OR smart-phone\* OR cellphone OR cell-phone OR cell-phones OR mobile-based OR "mobile tech\*" OR iphone\* OR i-phone\* OR blackberry\* OR black-berr\* OR ( ( smart OR mobile\* OR cell OR cellular OR portable OR transportable OR trans-portable OR wireless OR android OR apple OR google OR pixel OR huawei OR lenovo OR "LG" OR motorola OR nokia OR samsung OR galaxy OR xiaomi ) NEAR/1 ( phone\* OR telephone\* OR tele-phone\* OR handset\* OR hand-set\* OR device OR devices OR device-based OR phablet\* ) ) OR ( ( mobile OR smartphone\* OR "smart phone\*" OR "smart telephone\*" OR android OR apple ) NEAR/2 ( app OR application\* ) ) ) | 127,667 |
| 3 | #1 and #2 | 4,393 |
| 4 | #1 and #2 Science Citation Index Expanded (SCI-EXPANDED) | 3,991 |
| 5 | #1 AND #2 and English (Languages) Science Citation Index Expanded (SCI-EXPANDED) | 3,929 |
| 6 | #1 AND #2 and English (Languages) and Meeting Abstract or Proceeding Paper or Book Chapters or Retracted Publication or News Item or Biographical-Item or Bibliography or Retraction (Exclude – Document Types) Science Citation Index Expanded (SCI-EXPANDED) | 3,545 |
| 7 | #1 AND #2 Emerging Sources Citation Index (ESCI) | 404 |
| 8 | #1 AND #2 and English (Languages) Emerging Sources Citation Index (ESCI) | 377 |
| 9 | #1 AND #2 and English (Languages) and Proceeding Paper (Exclude – Document Types) Emerging Sources Citation Index (ESCI) | 375 |
| 10 | #6 OR #9 | 3,920 |
